# Effect of Welding Speed and Post Quenching on the Microstructure and Mechanical Properties of Laser-Welded B1500HS Joints

**DOI:** 10.3390/ma13204645

**Published:** 2020-10-18

**Authors:** Muyu Li, Dan Yao, Yingping Guan, Yongchuan Duan, Liu Yang

**Affiliations:** 1 Key Laboratory of Advanced Forging & Stamping Technology and Science, Yanshan University, Ministry of Education of China, Qinhuangdao 066000, China; Limuyu@stumail.ysu.edu.cn (M.L.); yaodanysu@163.com (D.Y.); yongchuan.duan@ysu.edu.cn (Y.D.); yangliu@ysu.edu.cn (L.Y.); 2 School of Mechanical Engineering, Yanshan University, Qinhuangdao 066000, China

**Keywords:** B1500HS high-strength steel, welding speed, microstructure, hardness, mechanical properties

## Abstract

In this research, B1500HS high-strength steel with different thicknesses were laser welded, and the effects of welding speed and post quenching were investigated by analyzing the microstructure, microhardness distribution, and high-temperature tensile properties of weld joints. The results show that an obvious difference can be found in the metallographic structure and grain morphology of the weld joint at different locations, which also lead to the significant uneven distribution of hardness. After quenching, the grain size of the original heat-affected zone was uniform, the columnar grains in the fusion zone were transformed into fine equiaxed grains, and no obvious hardness difference can be found in the weld joint. For the weld joint without quenching, the increase of welding speed can reduce the dimensions of grains of fusion zone and coarse grain zone, and slightly increase the hardness of these regions. In contrast, welding speed change has little influence on the microstructure and hardness of the weld joint after quenching. The high-temperature flow stress–strain curves of fusion zone welded under different welding speeds were calculated based on the mixture rule. The analysis results indicated that the fusion zone has higher strength but lower elongation than the base metal. In addition, the change of welding speed has a small impact on the high-temperature tensile properties of the fusion zone.

## 1. Introduction

As the energy crisis and environmental issues become increasingly prominent, lightweight technology has been continuously developing in automobile manufacturing. The light weight of vehicles can not only reduce fuel consumption and emissions, but also save on manufacturing costs [1,2]. The hot stamping process and forming process of tailor welded blank (TWB) are effective methods to realize the purpose and have been widely used in automobile parts production. In recent years, high-strength steel such as B1500HS has been used in the hot stamping process. During the forming process, the original microstructure of the sheet transforms into martensite, which can increase the strength of the material, thus reduce the usage of metal used in the product. At present, the hot stamping process of B1500HS has been used in the production of safety parts of automobile such as B-pillar and roof frame structure [3,4,5]. Tailor welded blank is comprised of sheets with different thickness or mechanical properties and joined by welding technology. The application of TWBs in the automotive industry can reduce material consumption, decreasing production costs, and improving local strength at the same time [6]. Through the combination of the forming process shown above, the application of B1500HS TWBs with unequal thickness in the hot stamping process can provide a more effective solution for the light weight of the car body.

The suitable welding method is of great significance to assure the weld quality of TWBs and satisfy production requirements. The commonly used welding methods include resistance spot welding, laser welding, gas shielded welding, laser welding, etc. Compared with the welding methods shown above, laser welding has many outstanding advantages, such as higher energy density, narrower weld, less distortion after welding, and faster welding speed [7,8]. Based on the benefits shown above, laser welding technology has been used in the production of TWBs.

High-strength steel laser welding technology has attracted scholars’ attention in recent years, and some achievements have been obtained. Windmann et al. [9] reported that the aluminum alloys in the coating melt into the fusion zone (FZ) of 22MnB5 TWB and form the brittle phase, which leads to the apparent decrease of mechanical properties of the weld joint. The research of Rossini et al. [10] found that the weld joint has low mechanical properties when the twinning induced plasticity steel is welded with other kinds of high-strength steel, which is due to the occurrence of intergranular fracture in the weld seam caused by the presence of Mn segregation. Some scholars [11,12] have explored the effect of laser welding on the microstructure and mechanical properties of quenching and partitioning steel and dual-phase steel, the softening zone inevitably appears in the heat-affected zone (HAZ) of both materials, which is attributed to the formation of tempered martensite and the softening zone is detrimental to the mechanical properties of the weld joint.

The change of welding process parameters can affect the performance of the weld joint, and hence the formability of TWB. Therefore, it is important to select appropriate welding parameters. For the laser welding process, the parameters that affect the quality of the weld joint include laser power, defocusing distance, flow rate of shielding gas and welding speed, etc. [13]. Gao et al. [14] studied the effect of welding speed on the microstructure of weld joint of dual-phase steel, and concluded that the increase of welding speed could reduce the size of austenite in FZ and obtain finer martensite. Sun et al. [15] explored the influence of heating input on the softening zone of laser-welded DP780, and the results show that the decomposition of martensite and precipitation of carbides is significant gradually with heat input increased, which result in the decrease of hardness in the softening zone. In order to reduce the softening effect caused by retained austenite and tempered martensite in HAZ, Alvea et al. [16] obtained the combination of process parameters with the minimum softening effect by adjusting the laser power and welding speed, thus improving the formability of TWB.

At present, many scholars have studied the laser welding of various kinds of high-strength steel from multiple perspectives. However, most of this research focuses on cold forming high-strength steel, such as dual-phase steel and twinning induced plasticity steel, with less concerns on high-strength steel used for hot forming. In addition, according to the characteristics of the hot stamping process, the workpieces should be fully austenitized at high temperature and quenched in die during the forming process. Existing research shows that the heat treatment process can affect the properties of laser-welded joints. Tang et al. [17] analyzed the microstructure of the B1500HS laser -welded joint before and after quenching and found that the columnar grains in the weld pool transformed into equiaxed grains with finer dimension after quenching. Luo et al. [18] investigated the effect of laser heat treatment on the property of laser-welded joints. The martensite in the weld pool and HAZ transformed into tempered martensite during the heat treatment, which makes the hardness of weld joints uniform, consequently improving the bending performance of the joints.

In this study, B1500HS high-strength steels with different thickness were welded by laser welding under various speeds. The effect of welding speed and post quenching on the microstructure, the distribution of hardness, and the mechanical properties of weld joints were analyzed. The research provides theoretical and practical guidance for the design of the laser welding procedure of B1500HS high-strength steel.

## 2. Materials and Methods 

### 2.1. Materials

**Figure 1:**
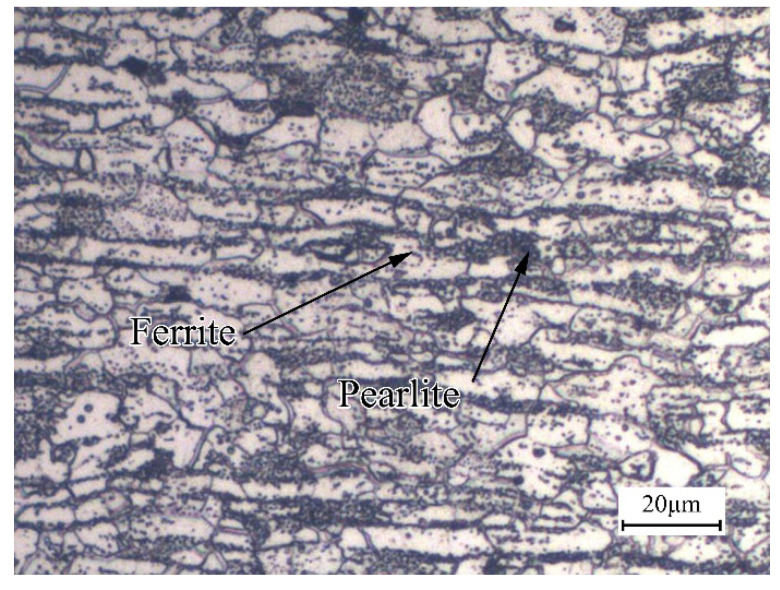
Microstructure of B1500HS high-strength steel.

The material used in the investigation is B1500HS cold-rolled high-strength steel, manufactured by the Baowu iron and steel group (Shanghai, China), and the thicknesses of the sheets are 1.2 mm and 1.6 mm. The chemical compositions of B1500HS are listed in Table 1, and the original microstructure of the base metal (BM) consists of ferrite and pearlite, as shown in Figure 1.

### 2.2. Laser Welding

**Figure 2:**
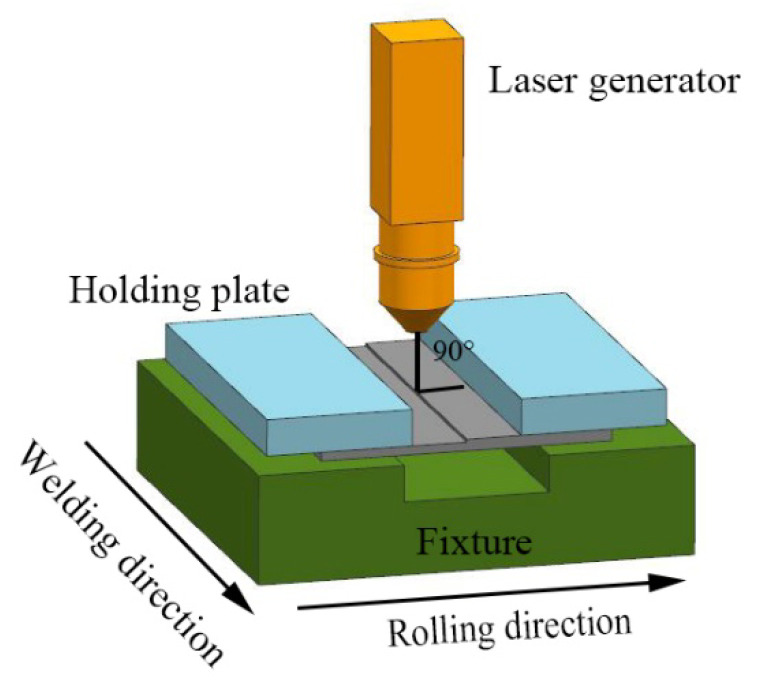
Schematic illustration of the welding process.

The sheets with different thickness were processed into blanks with dimensions of 200 mm × 80 mm. Before assembling, the blanks were polished with sandpaper to remove the corroded and oxide surface and then cleaned by acetone and anhydrous ethanol to eliminate the contaminant on the surface. The laser welding experiments of B1500HS TWBs were performed by the YLS4000 fiber laser system (IPG Photonics Corporation, Oxford, MA, USA). During the welding process, shielding gas has been used to stabilize the welding process and protect the welded seam against oxidization. The schematic illustration of the welding process is shown in Figure 2. The rolling direction of the BM was perpendicular to the welding direction, and the blanks with different thicknesses were held stationary with no gap in the fixture. The direction of shielding gas flow was set 45° to the welding direction, and the laser beam was perpendicular to the blanks. The laser welding parameters used in the experiment are listed in Table 2.

### 2.3. Microstructure

Metallographic specimens were cut from the cross-section of weld joints. In order to explore the effects of welding speed on the microstructure of weld joints before and after quenching, the specimens were divided into two groups. The specimens in group one were heated to 900 °C by a heating furnace and water quenched after soaked for 5 min, while the specimens in the other group were used as a control group without heat treatment. All specimens were mounted, grounded, polished, and etched by saturated picric acid in a 75 °C water bath and 4% nital solution to obtain the grain morphology and metallographic structure of weld joints. The grain morphology reflects the shape and size of grains, while the metallographic structure reflects the morphology and composition of metal metallography. The etched specimens were observed by an optical microscope. 

### 2.4. Mechanical Properties Tests

**Figure 3:**
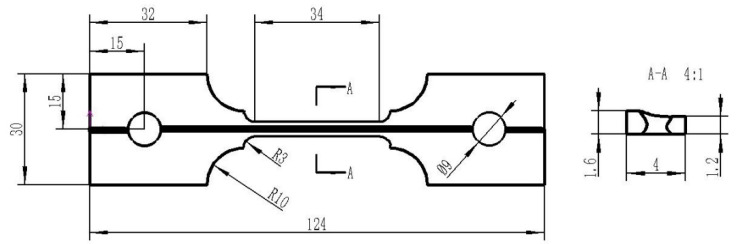
Geometry and dimensions of tensile samples.

High-temperature tensile tests were conducted to explore the effects of welding speed on the tensile properties of weld joints. The tests were carried out with a electronic universal tester and electric heating furnace (Chanxin testing machine manufacturing limited company, Jinan, China). The specimens were heated to 900 °C and held for 5 min to ensure austenitized completely. Afterward, the tensile tests were carried out with a strain rate of 0.05 s^−1^. The samples were cut from TWBs in the direction parallel to the weld line. Figure 3 shows the geometry and dimensions of the tensile samples. The tests were repeated three times to verify the accuracy of the measurement data. For comparison, the tensile properties of the BM perpendicular to the rolling direction were also measured under the same deformation conditions. 

According to GB/T4340.1-2009, the Vickers hardness tests were performed on the cross-section of etched weld joints using an FM-ARS9000 microhardness tester (FUTURE-TECH, Tokyo, Japan), with a load of 200 g and the holding time is 15 s. The hardness of the weld joint was measured along the rolling direction, and the space between measuring points was 0.1 mm. A hardness map of the entire weld joint was obtained based on the measured data.

## 3. Results and Discussion

### 3.1. Joint Appearance and Dimension

The change of welding speed has a significant impact on the welding quality. Defects such as burn through and incomplete penetration can be found when the selection of welding speed is not appropriate. By observing the TWBs welded under different welding speeds, weld joints with good quality can be achieved when the welding speed is between 18 and 50 mm/s. 

**Figure 4:**
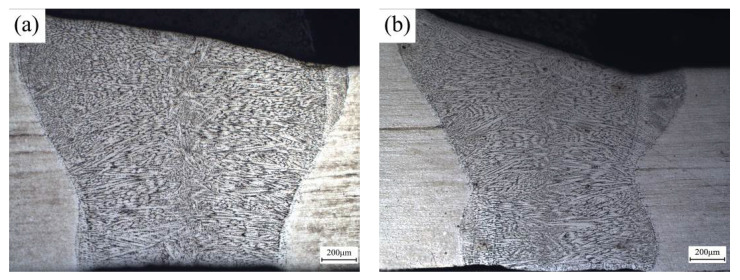
Metallographs of the cross-section of the weld joint under different welding speeds (**a**) 22 mm/s; (**b**) 40 mm/s.

After etched by saturated picric acid, the shape of FZ can be observed by an optical microscope (Carl Zeiss AG, Analytik Jena, Germany). Figure 4a,b show the metallographs of FZ welded by different welding speeds. It can be concluded that the FZ transits smoothly from the thick side to the thin side, and no welding defects such as gas hole, inclusion, and crack can be observed. In addition, the inclination angle of the upper surface of FZ decreases with the reduction of welding speed. A previous study indicated that appropriate reduction of welding speed is beneficial to the smooth transition of sheet metal on both sides of the weld line, which can improve the formability of TWB [19].

**Figure 5:**
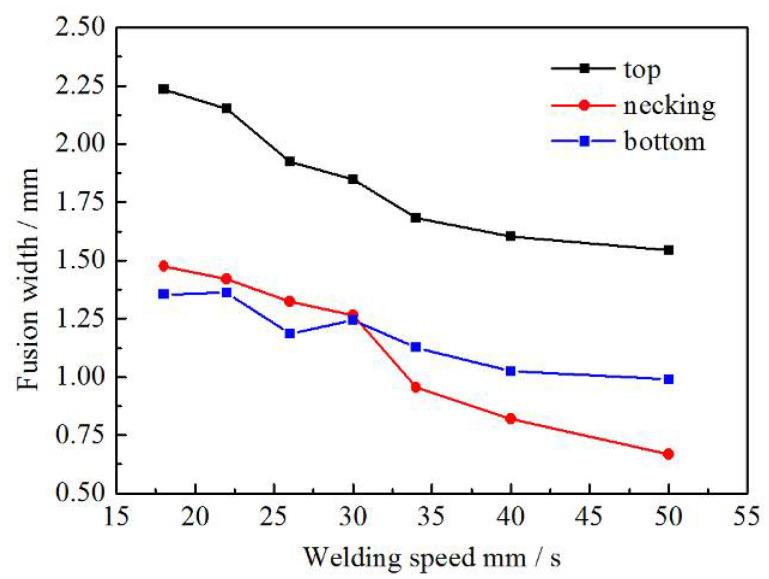
Relationship between the width of FZ and welding speed.

By observing the metallographs of weld joints, it can be found that the FZ presents an hourglass shape. Image processing software Image-Pro Plus (6.0) has been used to measure the width of FZ at the top, necking, and bottom position. Figure 5 shows the relationship between the width of FZ and the welding speed. It can be concluded that the width of FZ in these three positions decreases with the rise of the welding speed, and the reduction is more obvious in the necking position. Behzad et al. [20] summarized previous research, which investigated the influence of welding speed on the geometry of the FZ. The research results obtained by many scholars have confirmed that the increase of welding speed can reduce the width of the FZ, which is consistent with the measurement results in this paper. This phenomenon can be attributed to the change of heat input due to the variations of welding speed. The approximate value of heat input can be calculated by Formula (1).

(1) q=ηIU/v

where q is the value of heat input, η is heat efficiency, I and U is current and voltage respectively, and v is welding speed.

It can be found that the heat input is inversely proportional to the welding speed. The value of heat input decrease with the rise of welding speed, which reduces the volume of molten metal and finally leads to the reduction of weld width.

### 3.2. Microstructure

#### 3.2.1. Microstructure of Weld Joint before Quenching

**Figure 6:**
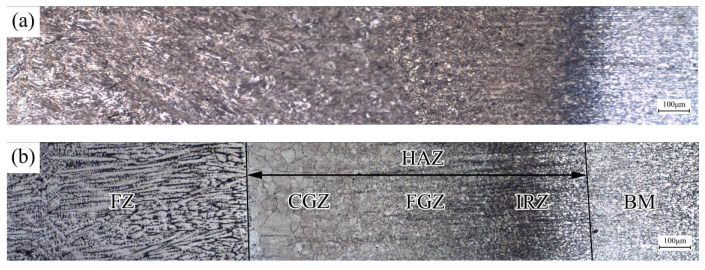
Distribution of (**a**) metallographic structure and (**b**) grain morphology of weld joint.

Figure 6a,b shows the distribution of metallographic structure and grain morphology of the B1500HS welded joint from the weld center to the BM. Significant evolution in microstructure can be observed across the weld joint. 

The laser-welded weld joint of B1500HS can be divided into three zones: FZ, HAZ, and BM. During the welding process, the metal in FZ was heated to a molten state and rapidly cooled to solidification. In previous research, the microstructure composition of FZ can be inferred according to the continuous cooling transformation diagram and cooling time $t_{T/T_{0}}$. The value of $t_{T/T_{0}}$ means the time required for the FZ cooling from high temperature T to low temperature $T_{0}$ after welding, which can be calculated by Equation (2) [21]:

(2) $$t_{T/T0}=\frac{\alpha }{4\pi \lambda^{2}}{\left(\frac{q}{vh}\right)}^{2}\left(\frac{1}{{\left(T_{0}-t\right)}^{2}}-\frac{1}{{\left(T-t\right)}^{2}}\right)$$

where α is the thermal diffusivity (heat conductivity/(density × specific heat)), λ is the thermal conductivity, q is the heat input, h is the thickness of the sheet, v is welding speed, and t is the preheating temperature before welding.

**Figure 7:**
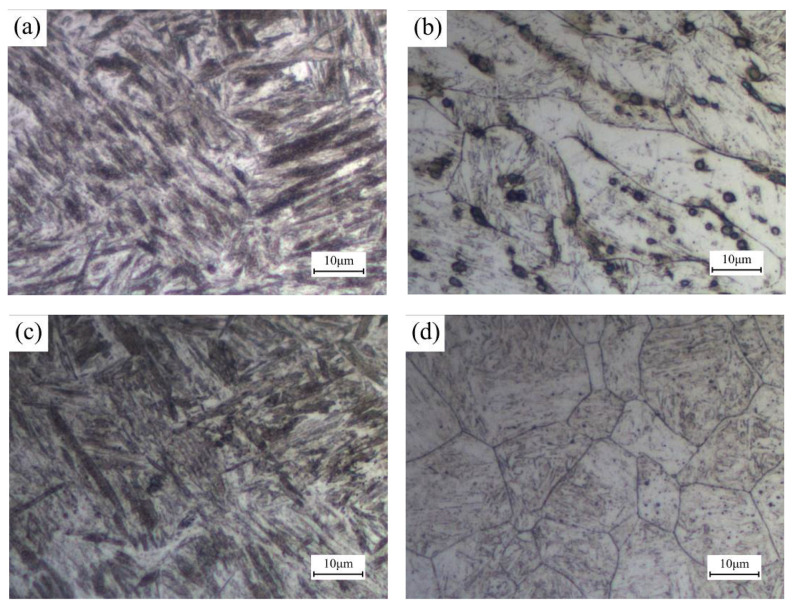

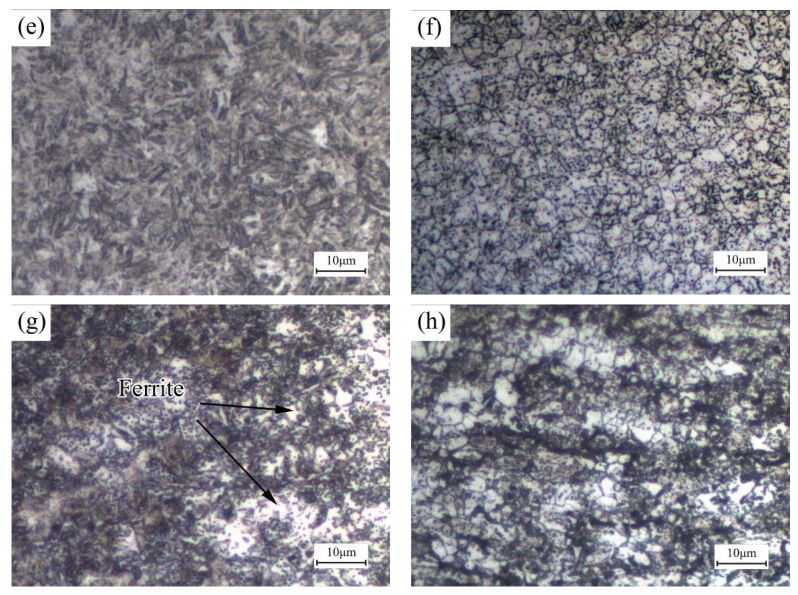
Metallographic structure of (**a**) FZ; (**c**) CGZ; (**e**) FGZ; (**g**) IRZ, and grain morphology of (**b**) FZ; (**d**) CGZ; (**f**) FGZ; (**h**) IRZ of weld joint welded under 30 mm/s.

For B1500HS high-strength steel, non-martensite transformation occurs in the temperature range from 900 °C to 400 °C. Thus, the cooling time $t_{9/4}$ of FZ under different welding speeds were calculated in this paper. After referring to the present research [22], the thermophysical parameters of B1500HS can be achieved. The value of heat input can be calculated by Formula (1), and the heat efficiency η is defined as 0.5 [23]. The cooling time $t_{9/4}$ can be calculated by substituting the above data into Formula (2). When the welding speed increases from 18 mm/s to 50 mm/s, the values of $t_{9/4}$ decrease from 7.13 s to 0.92 s. It indicated that the increase of welding speed can increase the cooling rate of FZ, which is significantly higher than the critical cooling rate of B1500HS [24]. Therefore, the microstructure composition of FZ is basically composed of martensite and a few retained austenite due to the incomplete transformation of martensite, as shown in Figure 7a.

During the welding process, the molten metal cools rapidly through the direction perpendicular to the fusion line. According to the competitive growth mechanism, the grains in FZ tend to grow along the cooling direction. Figure 7b shows the grain morphology of FZ, and columnar grains can be observed. In addition, the rapid cooling rate leads to the uneven distribution of chemical compositions in this region, which leads to the occurrence of segregation.

In the HAZ, the thermal cycle changes with the distance to the heat source, which affects the transformation of the microstructure of the HAZ. According to the distinct in the grain morphology and composition of microstructure, the HAZ can be divided into coarse grain zone (CGZ), fine grain zone (FGZ), and incomplete recrystallization zone (IRZ).

Since the heating temperature of CGZ and FGZ was above $A_{c3}$, the metals of the two regions were fully austenitized in the welding process and transformed into martensite in the subsequent rapid cooling process. The CGZ is adjacent to the FZ, the peak temperature of the thermal cycle experienced in the welding process is between the melting point, and the superheat temperature, which leads to the austenite grains of CGZ growing seriously, especially near the fusion line. Compared with the CGZ, the metal of FGZ was heated to a lower temperature, ranging from superheat temperature to $A_{c3}$, which decreases the growth tendency of grains in this region. The grain morphology of CGZ and FGZ is shown in Figure 7d,f, respectively. It can be observed that the grain size of FGZ is much smaller than that of CGZ. Fine austenite grains can limit the dimension of martensite. By comparing the metallographic structure shown in Figure 7c,e, finer martensite can be found in FGZ. 

For the IRZ, the peak temperature here ranged from $A_{c3}$ to $A_{c1}$, and only part of the original microstructure transformed into austenite during the welding process, and transformed into martensite in the following quenching process. As a consequence, the microstructure composition of IRZ is a combination of ferrite and martensite, as shown in Figure 7g. 

#### 3.2.2. Microstructure of Weld Joint after Quenching

**Figure 8:**
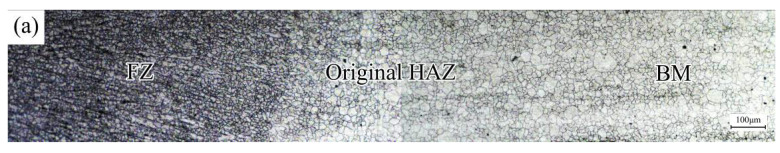

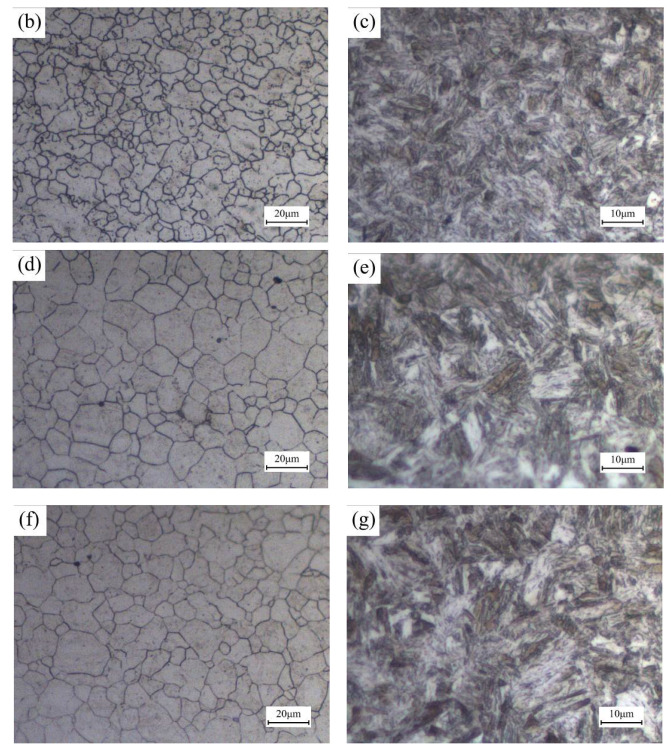
Grain morphology of quenched weld joint (**a**) cross-section; (**b**) FZ; (**d**) original HAZ; (**f**) BM, and the metallographic structure of (**c**) FZ; (**e**) original HAZ; (**g**) BM.

Figure 8a shows the distribution of grain morphology of the B1500HS weld joint after quenching. It can be observed from the figure that the grain morphology of FZ is obviously different from that of other positions in the weld joint. By comparing the grain structure of original HAZ and BM shown in Figure 8d,f, it can be concluded that these regions are composed of uniform equiaxed grains with similar grain size. This phenomenon is the combination effect of recrystallization and grain growth during the heating process. In contrast, the heat treatment has more obvious grain refinement effect on FZ, as shown in Figure 8b. The original columnar grains were transformed into equiaxed grains, which are finer than those of original HAZ and BM. In addition, it can also be found that the grain size of FZ is not uniform and many irregular grains can be observed. 

Figure 8c,e,g shows the metallographic structure of quenched weld joint in different regions. Through the comparison and analysis, it can be found that the FZ has smaller martensite compared with other regions, which is due to the limitation of grain size on the growth of martensite. For the same reason, the dimension of martensite in the BM is similar to that in the original HAZ.

#### 3.2.3. Effect of Welding Speed on the Microstructure of Weld Joint

**Figure 9:**
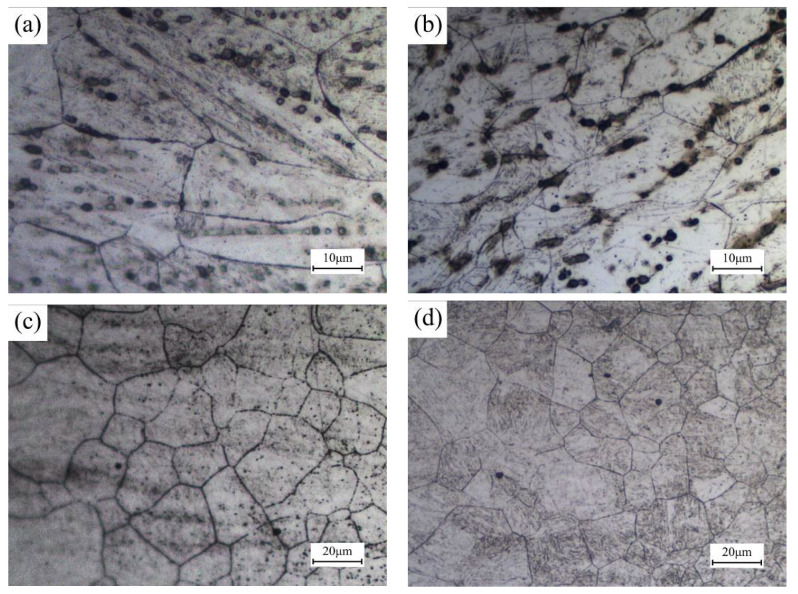
Grain morphology of FZ (**a**) welding speed 22 mm/s; (**b**) welding speed 40 mm/s, and grain morphology of CGZ (**c**) welding speed 22 mm/s; (**d**) welding speed 40 mm/s.

Figure 9a–d shows the grain morphology of FZ and CGZ before quenching under the welding speeds of 22 mm/s and 40 mm/s. For these two regions, the increase of welding speed can decrease the dimension of grains. According to Formula (2), the rise of welding speed can reduce cooling time, which reduces the residence time of metal at high temperature. In addition, the peak temperature of thermal cycle in FZ and CGZ was higher than superheat temperature, which induced the rapid growth of grains in these regions. Therefore, finer grain size can be observed in FZ and CGZ with the increase of welding speed, which is also beneficial for obtaining finer martensite. 

Because of the grain size of the FGZ and IRZ is very small and growth tendency is weak due to the peak temperature lower than the superheat temperature, the limited decrease of cooling time caused by the increase in welding speed is not enough to produce an obvious refining effect on the grain morphology of FGZ and IRZ. Because the microstructure of IRZ changes significantly in the narrow region, the effect of welding speed on the microstructure composition of IRZ cannot be analyzed objectively by comparing the metallographic diagrams of IRZ under different welding speeds. However, the rise of welding speed can increase the cooling rate, thus reducing the formation of non-martensite structure. However, the enhanced cooling effect was not obvious in IRZ. Hence, the influence of welding speed on the microstructure composition of IRZ is not obvious. Based on the analysis above, the change of welding speed has no apparent influence on the microstructure of FGZ and IRZ.

**Figure 10:**
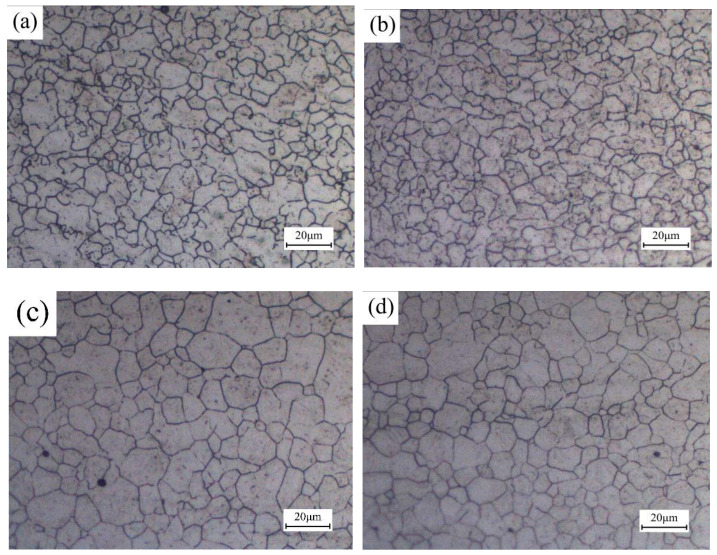
Grain morphology of FZ (**a**) welding speed 22 mm/s; (**b**) welding speed 40 mm/s, and grain morphology of original HAZ (**c**) welding speed 22 mm/s; (**d**) welding speed 40 mm/s after quenching.

The analysis results shown in Section 3.2.2 indicate that the grain morphology of HAZ becomes uniform during the heating process. Figure 10a–d shows the grain morphology of FZ and original HAZ after quenching, which were welded under different welding speeds. It can be concluded that the welding speed has no noticeable effect on the grain morphology of quenched weld joints. During the heating process, the occurrence of recrystallization and grain growth eliminate the difference of grain morphology caused by the change of welding speed before heat treatment.

### 3.3. Microhardness

**Figure 11:**
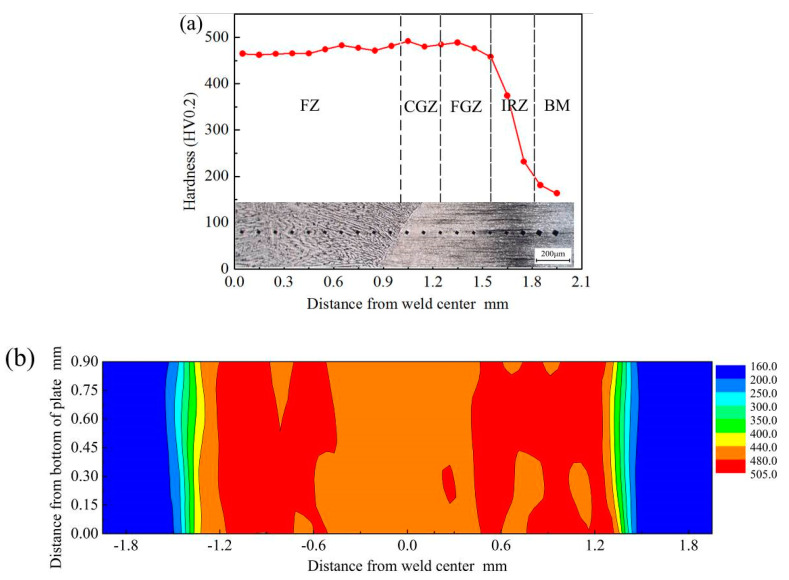
Hardness evolution (**a**) hardness distribution and grain morphology and (**b**) hardness map of the cross-section of the weld joint.

Hardness can be used to evaluate the anti-deformation ability of metal. Figure 11a shows the hardness distribution of the laser-welded B1500HS joint from the center of FZ to the BM. In this research, the distribution of hardness was analyzed by combining the grain morphology of the corresponding position. It can be found that the hardness of FZ, CGZ, and FGZ is significantly higher than that of BM. After the welding process, the microstructure of these three regions is mainly composed of martensite, which leads to the obvious increase of hardness. In addition, the value of hardness slightly increases from FZ to FGZ, which is mainly caused by the difference of grain size between regions. Fine grain size can enhance the effect of fine-grained strengthening, thus the hardness of FGZ is slightly higher than that of CGZ and FZ. A dramatic slump can be observed in IRZ, which is caused by the existence of non-martensite such as ferrite. 

Figure 11b shows the hardness map of the laser-welded B1500HS joint. It can be concluded that only slight changes in hardness can be observed in the direction vertical to the sheet. Along the rolling direction, the change rule of hardness is identical with the curve shown in Figure 11a.

**Figure 12:**
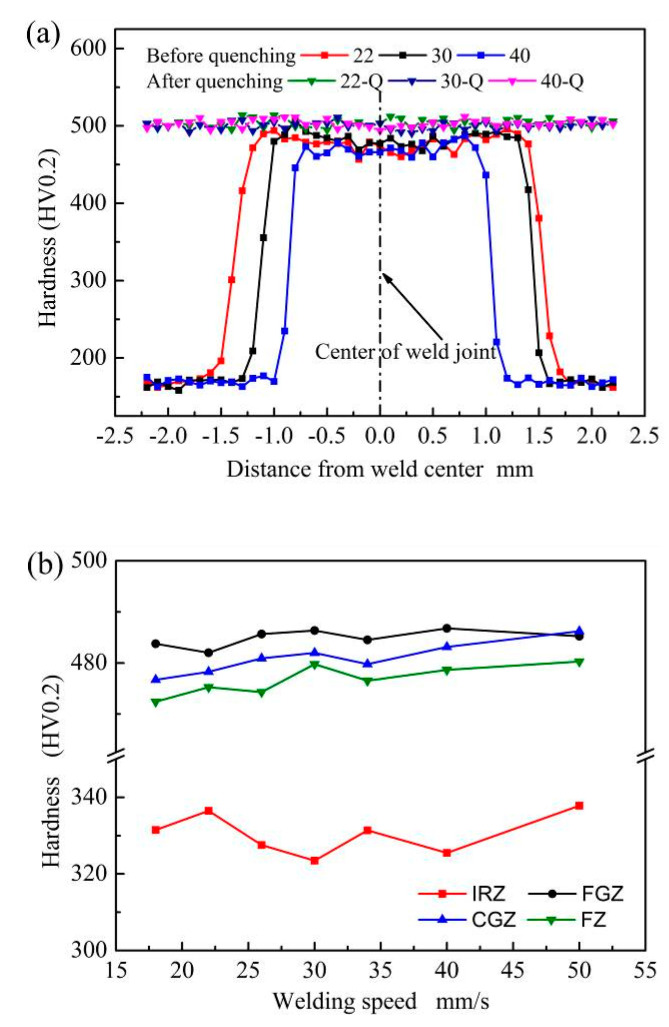
Effect of welding speed on the hardness of weld joint (**a**) hardness of weld joint before and after quenching; (**b**) relationship between the average hardness of different regions of welded joints without quenching and welding speeds.

Figure 12a shows the hardness distribution curves of weld joints under different welding speeds before and after quenching. For the weld joints without heat treatment, the increasing of welding speed can reduce the width of the area with high hardness, which is composed of FZ, CGZ, and FGZ.

In order to investigate the influence of welding speed on the hardness of the laser-welded B1500HS joint in different regions, the average hardness values of each region were calculated and shown in Figure 12b. The average hardness of FZ and CGZ slightly increased with the rise of welding speed, but this trend is not obvious in FGZ. Compared with FGZ, the grain size of FZ and CGZ decrease more obviously with the rise of welding speed, which slightly enhances the effect of fine-grained strengthening, resulting in the small increment of hardness in these two regions. In addition, it can be found that the average hardness values of IRZ fluctuate irregularly with the increase of welding speed. The hardness values of IRZ change obviously in a small width, making it uncertain to calculate the average hardness of IRZ. 

Figure 12a also shows the hardness distribution of quenched weld joints, and no noticeable difference in the hardness can be observed at different positions of the weld joints. The microstructure of the whole quenched weld joint is composed of martensite and a small amount of retained austenite, which is the primary reason for the similar hardness in different positions of the weld joint. Compared with other regions, the quenched weld joint has finer grain size in FZ, but the difference in grain size is much smaller than that between the FGZ and FZ of weld joint without heat treatment. Thus, the difference of grain morphology between FZ and other places did not cause the obvious difference in hardness. 

By comparing the hardness distribution of quenched weld joints welded by different welding speeds, it can be concluded that the welding speed has no pronounced effect on the hardness of weld joints after quenching.

### 3.4. Tensile Properties at High Temperature

**Figure 13:**
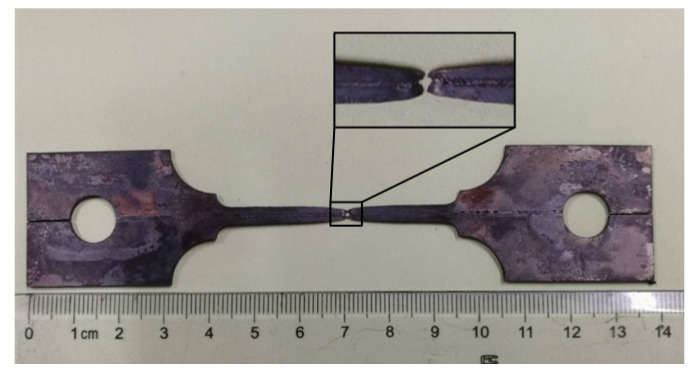
Fracture sample after tensile at high temperature.

Figure 13 shows the photograph of the fractured tensile sample of laser-welded B1500HS. By observing the fracture position of the specimen, it can be found that the fracture is V-shaped, which indicates that the FZ cracked first during the tensile process. 

During the heating process, the microstructure of the whole weld joint transformed into austenite. According to the analysis results shown in Section 3.2.2, the grain morphology in different positions of the weld joint is not the same. The original HAZ has uniform equiaxed grains similar to the BM, while the grain size of FZ was finer than that of BM, but the dimension and shape of grains were not uniform. In the case of the same microstructure composition, the difference of grain morphology can lead to the diversity of tensile properties. 

**Figure 14:**
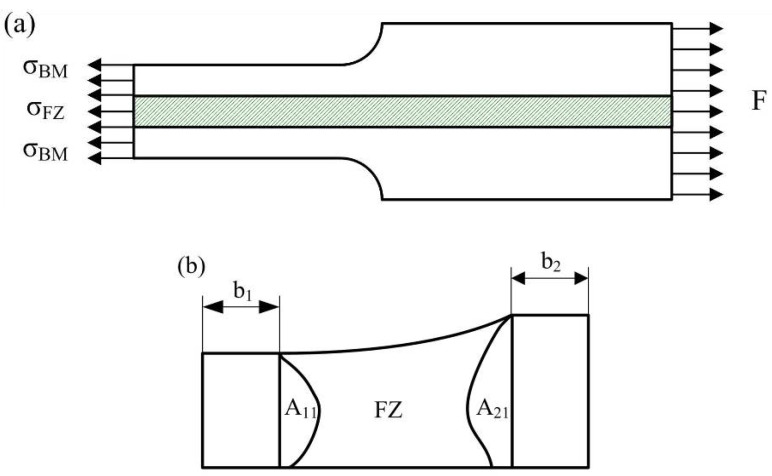
Schematic diagram of (**a**) the uniaxial tensile mechanical model of TWB sample and (**b**) the cross-section of tensile sample.

In the previous research, a mixed method has been used by scholars to calculate the tensile properties of the weld joint [25,26,27]. In this study, the flow stress-strain curves of FZ at high temperatures were calculated by the mixture method. The mechanical model of B1500HS TWB under tensile test is shown in Figure 14a. 

According to the previous analysis of the microstructure of the welded joint at high temperature, it is considered that the original HAZ has the same tensile properties with the BM. In contrast, the tensile properties of FZ is different from that of other regions. In the mixture rule, the flow stress-strain curves of FZ can be calculated from the force-displacement curves measured during the tensile test, considering the force equilibrium and equal strain condition between the FZ and other places. The flow stress of FZ can be expressed as the following formula:

(3) $$\begin{array}{l} \sigma_{FZ}=\frac{F-\sigma_{BM}A_{BM}}{A_{FZ}}\\ A_{BM}=A_{0}{}_{BM}\times L_{0}/\left(L_{0}+\Delta L\right)\\ A_{FZ}=A_{0}{}_{FZ}\times L_{0}/\left(L_{0}+\Delta L\right) \end{array}$$

where F denotes the tensile force measured during the test, A, $A_{0}$ and σ are the instantaneous area, initial area, and flow stress, respectively, $L_{0}$ and ΔL are initial gauge length of sample and displacement of the testing machine.

**Figure 15:**
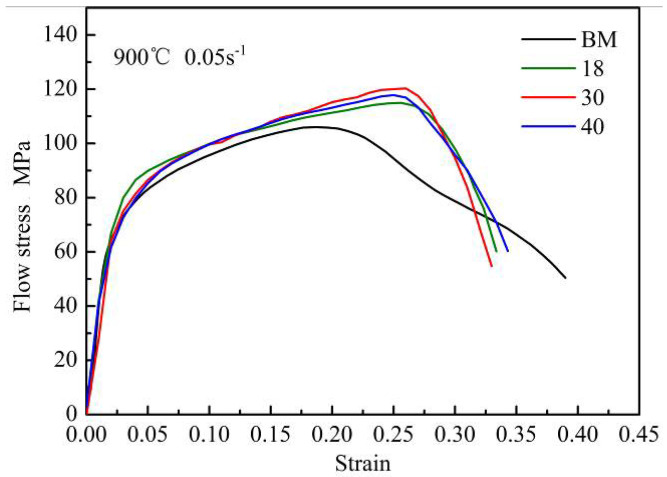
Flow stress-strain curves of the FZ and BM.

During the calculation of the flow stress-strain curve of FZ, the measurement of the initial area $A_{0\;BM}$ and $A_{0\;FZ}$ has a significant impact on the accuracy of the calculation results. Figure 14b shows the schematic diagram of the cross-section of the tensile sample. After etched by saturated picric acid solution, the shape of FZ can be observed by optical microscope, and the value of $A_{0\;FZ}$ can be measured from the metallograph by image processing software. The area of $A_{11}$ and $A_{12}$ shown in Figure 14b can also be obtained with the same method. The value of $A_{0\;BM}$ can be obtained by the following formula:

(4) $$A_{0\;BM}=1.2\times b_{1}+A_{11}+A_{12}+b_{2}\times 1.6$$

where $b_{1}$ and $b_{2}$ is the width of BM with different thicknesses, which can be measured from the upper surface of the gauge section. By substituting the relevant data into Formula (3), the flow stress of FZ can be obtained. The flow stress-strain curves of FZ welded under different welding speeds were compared with that of BM in Figure 15.

It can be found that the flow stress-strain curve of FZ is obviously different from that of BM. The flow stress-strain curve of the BM decreases twice with the increase of strain. According to the results of previous research [28], dynamic recrystallization occurs in B1500HS high-strength steel under high temperature and low strain rate conditions. The softening effect caused by this phenomenon leads to the flow stress of the BM decrease for the first time with the increase of strain. Meanwhile, the second drop of flow stress-strain curve is caused by the material instability. In contrast, the flow stress of FZ still keeps the upward trend when the flow stress of the BM decreases due to the softening effects. In addition, it can also be concluded that the flow stress of FZ is higher than that of BM under the same strain condition. During the heating process before quenching, the grain morphology of weld joint changes obviously, and the grain size in FZ is smaller than that in BM. The increase of grain boundary has a greater hinder effect on the movement of dislocation, thus improving the strength of FZ. By comparing the flow stress-strain curves of FZ welded under different welding speeds, it can be concluded that the change of welding speed has limited influence on the tensile properties of FZ.

Table 3 shows the elongation of B1500HS weld joints and BM. It can be found that the elongation of the BM is significantly higher than that of the weld joints, and the change of welding speed has no noticeable effect on the elongation of FZ. Although the heating process can obviously refine the grains in FZ, the grain size is not uniform and many grains have an irregular shape, which leads to the non-uniform distribution of deformation between grains in FZ, resulting in the local stress concentration and promote the initiation and propagation of cracks. During the high-temperature tensile process, the FZ of the samples cracked first, and then extended to both sides of the BM with the increase of deformation, resulting in the fracture of sample showing a V-shape.

## 4. Conclusions

In this paper, the effect of welding speed and post quenching on the microstructure and mechanical properties of the B1500HS weld joint has been investigated. The following conclusions were derived from the experimental results:

1. The increase of welding speed can improve the cooling rate of the weld joint and obtain finer grains in FZ and CGZ.

2. For the quenched weld joint, the grain morphology of the original HAZ becomes uniform and the grain size is similar to that of BM. In addition, the original columnar grains in FZ transformed into equiaxed grains finer than that of BM, but the grain dimension is not uniform and many grains are irregular in shape.

3. With the increase of welding speed, the hardness of FZ and CGZ increases slightly. For the quenched weld joint, the hardness values of the welded joint at different positions were uniform and basically the same. In addition, the change of welding speed has no obvious effect on the hardness of the welded joint after quenching.

4. The flow stress-strain curves of FZ welded under different welding speeds were calculated based on the mixture method. Compared with the BM, the FZ has higher strength but lower elongation. Furthermore, the change of welding speed has no significant effects on the tensile properties of FZ at high temperatures.

## Figures and Tables

**Table 1:** Chemical composition of B1500HS steel (wt %).

C	Si	Mn	Cr	Ni	Mo	B	Al	Ti	Cu	V	S	P
0.23	0.25	1.35	0.19	0.028	0.04	0.003	0.04	0.03	0.016	0.004	0.006	0.015

**Table 2:** Laser welding parameters used in the experiment.

Laser power	2.5 kW
Flow rate of shielding gas	15 L/min
Welding speed	18, 22, 26, 30, 34, 40, 50 mm/s
Defocusing distance	0 mm
Spot size	0.4 mm

**Table 3:** Elongation and peak stress of base metal and FZ under different welding speeds.

Welding Speed mm/s	Elongation
18	46.54%
30	42.18%
40	45.43%
BM	54.85%
